# Supplementary material for: Prevalence of specific micronutrient deficiencies in urban school going children and adolescence of India: A multicenter cross-sectional study
Source: PLoS One. 2022 May 11;17(5):e0267003. doi: 10.1371/journal.pone.0267003 (PMC9094555; doi:10.1371/journal.pone.0267003)
Supplement: Attachment — Submitted filename: Response to Comments.pdf [file pone.0267003.s001.pdf]

Response to comments

Sr. No.	Comment	Response
1	Please note that you must upload a completed CONSORT flowchart as figure 1 of your manuscript. Blank copies of this document and information regarding CONSORT can be found via the following link: http://www.consort-statement.org/.	Dear Editor, the current study is a cross- sectional observational study. It is not a randomized control study or a clinical trial. Hence, CONSORT is not applicable.
2	Please note that you must upload a completed CONSORT checklist as a supporting information file. Blank copies of this document and information regarding CONSORT can be found via the following link: http://www.consort-statement.org/. If your clinical trial uses a non-randomized design, you may wish to submit a TREND checklist (http://www.cdc.gov/trendstatement), in place of the CONSORT checklist.	Clarified in point no 1.
3	Please upload a copy of your trial study protocol as a supporting information file. By the study protocol, we mean the complete and detailed plan for the conduct and analysis of the trial that the ethics committee approved before the trial began. Please send this in the original language. If this is in a language other than English, please also provide a translation. Please detail any deviations from this study protocol in the Methods section of your manuscript. Your study protocol will be made available to the editors and reviewers, and will be published as supporting information with your manuscript if accepted for publication. (If you do not agree to this, we will not be able to publish your manuscript). If you have formally published a study protocol for your trial in a journal then you should cite this in your manuscript, but you still need to send us the original document.	The study protocol was submitted for publication to BMJ Open and is currently under review. The submitted manuscript ID is bmjopen-2020-046783.R1.<br><br>We have uploaded a copy of protocol.
4a	Please provide the complete date range for participant recruitment and follow-up in the methods section of your manuscript.	As mentioned in point no. 1, this is a cross-sectional observational study. No follow up of study participants was done.<br>However, enrollment of participants was done from April 2019 to February 2020 (pg. no. 9, line no. 187). Data collection, including biochemical analysis of samples lasted from April 2019 to March 2021 (pg. no. 6, line no. 122).
4b	If you have not yet registered your trial in an appropriate registry, we now require you to do so and will need confirmation of the trial registry number before we can pass your paper to the next stage of review. Please include in the Methods section of your paper your reasons for not registering this study before enrolment of participants started. Please confirm that all related trials are registered by stating: “The authors confirm that all ongoing and related trials for this drug/intervention are registered”.	The study was registered prospectively, with Clinical Trial Registry of India (CTRI). Registration number is CTRI/2019/02/017783. CTRI is online public record system for registration of clinical trials and other studies being conducted in India, involving human participation.<br>It is mentioned in abstract (pg. no. 3, line no. 51) and method section (pg. no. 4, line no. 83).<br>We confirm that this study is not a clinical trial and did not involve any drug, device or intervention.
