# Supplementary material for: Prevalence of specific micronutrient deficiencies in urban school going children and adolescence of India: A multicenter cross-sectional study
Source: PLoS One. 2022 May 11;17(5):e0267003. doi: 10.1371/journal.pone.0267003 (PMC9094555; doi:10.1371/journal.pone.0267003)
Supplement: Attachment — Submitted filename: Response to Reviewers Comments 2.docx [file pone.0267003.s002.docx]

Response to comments

| Sr. No. | Comment | Response | Page No. | Line No. |
| --- | --- | --- | --- | --- |
| 1 | We note that you have stated that you will provide repository information for your data at acceptance. Should your manuscript be accepted for publication, we will hold it until you provide the relevant accession numbers or DOIs necessary to access your data. If you wish to make changes to your Data Availability statement, please describe these changes in your cover letter and we will update your Data Availability statement to reflect the information you provide | All the relevant data has been included in the manuscript. However, if data is required data will be available on request. |  |  |
| 2 | Abstract  I don’t think the sentence “In India, around 0.5 per cent of total deaths in 2016 were contributed by nutritional deficiencies.” Is important to justify the study | Removed as advised by esteemed reviewer. |  |  |
| 3 | Background:  Line 67-69: please cited the surveys reported here. | Done as advised. Reference numbers 12 – 16. | 4 | 74-81 |
| 4 | Background:  metal >> mineral | Done as advised | 2  3  4 | 33  61  90 |
| 5 | Background:  The purpose of the study is to fill the scarcity of a nationally representative data on micronutrient deficiencies but the actual study was limited to urban areas. Please, reorganize the purpose of the study accordingly. | Yes, we agree with the esteemed reviewer. This study was limited to urban areas. We had written in background section “The aim of this current study is to fill this knowledge gap by collecting data through a well-planned, large-scale study using standardized methodologies to assess the prevalence of various micronutrient deficiencies, among urban school going children. The primary objective of study was to assess the prevalence of deficiencies of Vitamins (Vitamin A, 25 Hydroxy Vitamin D, Vitamin B12 and Folate) and minerals (Calcium, Zinc, Selenium and Iron), among urban school going children aged 6-11 and 12-16 years in ten cities of India. Our secondary objective was to find the association between micronutrient deficiencies with sociodemographic and anthropometric indicators.” We had written this in manuscript title as well.  This is a limitation of the study and has been written in discussion. | 4  16 | 86 – 93  347 |
| 6 | Methodology  Please justify why these specific micronutrients were selected for the study. | It is now written in the manuscript as “Vitamins and minerals, termed together as micronutrients, although required in small quantities, are vital for the normal growth, development and functioning of human body. These are essential throughout the life but the period of childhood and adolescence is more important, as it is marked by rapid growth and development. Review of literature suggests that deficiency of minerals (calcium, iron, selenium and zinc) and Vitamins (Vitamin A, 25 Hydroxy Vitamin D, Vitamin B12 and Folate) during childhood and adolescence have negative impact on general health, growth, neuropsychological behavior, cognitive and motor development, intelligence quotient, attention, learning, memory, language ability and educational achievement [1-9]. Iron, folate or vitamin B12 deficiency results in anaemia which has a negative impact on work capacity, intellectual performance, and child cognitive development [1]. Vitamin A plays a critical role in eye health and immune function [1]. Zinc deficiency can interfere with multiple organ systems including brain development and cognition, particularly when it occurs during a time of rapid growth and development [2].” | 3 | 58-70 |
| 7 | Line 85: how were these ten major cities selected? How do you assure the representativeness of the sample for the urban population of the country? | We have prepared a list of institutions across the country, involved in conducting community based high quality research with KGMU and send them invite to participate. Those who consented, participated in the study.  While selecting we ensured that there were districts from central (Lucknow, Patna), northern (Srinagar, Chandigarh), southern (Bangalore, Bhubaneswar, Manipal and Thiruvananthapuram), eastern (Dibrugarh) and western (Jodhpur) parts of the country. By this we ensured representativeness of the samples for urban population of the country.  Detailed study protocol is published elsewhere (Ref. no. 30) |  |  |
| 8 | Line 86: Each school contributed equal sample size (about 240). Why it was not possible to take the size of each school into consideration? Or at analysis level, weighted analysis, based on the student size of the schools, should be done. | As per the multicentric study protocols and convention, whole data is considered as one unit for analysis. If site investigators want some information, they may do separate analysis for their district. However, this will not have enough power. If we do weighted analysis, purpose of multicentric analysis is lost. That is why 240 children were taken from each district. |  |  |
| 9 | Sample size was estimated based on folic acid deficiency? The adequacy of the available sample size for measuring the prevalence of other micronutrient deficiencies should also be tested. | Here we have calculated the sample sizes for other micronutrient deficiencies also and have picked the one (corresponding to folate deficiency) which gave maximum sample size. This has now been written in the manuscript as “The sample size was calculated using folate deficiency as this gave the maximum sample. Assuming the prevalence of folate deficiency in India as 30.7% [32], precision (d) of 2% and level of significance (α) 0.05, the calculated sample size [33] was 2044 participants. After taking, attrition rate of 10% sample size inflate to 2400 participants.” | 5 | 113-116 |
| 10 | Why was the study limited to students living within 5 km radius of the school? Please also discuss the possible implication of this approach (possible selection bias?). | The 5 km distance sampling frame was chosen considering logistic and time issues, as other component (3-day dietary intake) of this study includes home visit by research team.  Detailed study protocol is published elsewhere (Ref. no. 30).  We agree that there could have been selection bias and have mentioned this as one of the draw backs of the study in the revised manuscript as “A selection bias was also possible as the current study included the participants residing within 5 km radius of the school.” |  |  |
| 11 | Line 115: BMI less than 12.5%??? BMI in %? Why you did not use BNI-for-age, which is more appropriate for adolescents? | As per the recommendations of the WHO expert committee for assessing anthropometric indicators, BMI for age below 12.5 is for the females aged 61 months, condition is considered as severe thinness (BMI for age < –3 SD of the WHO Child Growth Standards median). Hence for uniformity we considered this. |  |  |
| 12 | Line 128: please briefly describe the “Kuppuswamy’s socioeconomic scale” | In India, Kuppuswamy’s scale is used to assess the socioeconomic status. It classifies the socioeconomic status into five categories – Upper, Upper middle, Lower middle, Upper lower and Lower class, based on the education and occupation of head and monthly family income (Reference no. 34). | 6 | 139 |
| 13 | Blood collection and processing: have you done anything to reduce external contamination of blood samples for trace minerals? This is especially important for zinc analysis. | Yes definitely, we have employed all the precautions to minimize the risk of trace metal contamination. Detailed study protocol is published elsewhere (Ref. no. 30) |  |  |
| 14 | Table 1:  please improve the formatting. Table 1 and 2 can also be merged. | Done as advised by esteemed reviewer. |  |  |
| 15 | The variables of the study (including those considered for the multilevel analysis) should be exhaustively described and listed before the data collection section. | Done as advised. It is now written as “To use the nested structure of children within school, multiple logistic regression model as well as individual level logistic model were used to find the associations of important factors (sociodemographic profile like age, gender, family type, mother’s education, socioeconomic class and anthropometric indicators like severe thinness/thinness, overweight, obese/severe obese, stunting) on outcome variable (micronutrient deficiency)” | 9 | 201 – 205 |
| 16 | Line 182-185: what were these “sociodemographic profile and anthropometric indicators” what was the specific dependent variable? Because you have analysed several micronutrients. | All the sociodemographic and anthropometric variables, as listed above in point no. 15, are independent variables. Among these, no dependent variable constitutes the predictor part. |  |  |
| 17 | Results  I don’t think table 3 is really important. As you described in the methods section, equal samples of boys and girls have been taken. So the balance observed between boys and girls is not surprising. | Removed as advised by esteemed reviewer. |  |  |
| 18 | In all tables, please only provide the frequency and %, the denominator should rather be provided at the top of the column. | Since the denominator for various variables presented in tables is different, hence they are provided for each variable. |  |  |
| 19 | How did you classify socio-demographic status into five? | In India, Kuppuswamy’s scale is used to assess the socioeconomic status. It classifies the socioeconomic status into five categories – Upper, Upper middle, Lower middle, Upper lower and Lower class, based on the education and occupation of head and monthly family income (Reference no. 34). |  |  |
| 20 | Table 4: I could not get the point of statistically comparing the sociodemographic and anthropometric characteristics of the two groups. Please remove the statistical testing. The sentence on line 195 and 196 should also be removed. | Done as advised by esteemed reviewer. |  |  |
| 21 | Please describe the micronutrient status under a separate sub-header. | Done as advised by esteemed reviewer. |  |  |
| 22 | Contents in table 5 should be concisely described before the table itself. | Done as advised by esteemed reviewer. |  |  |
| 23 |  |  |  |  |
| 24 | 1. Is the manuscript technically sound, and do the data support the conclusions?  The manuscript must describe a technically sound piece of scientific research with data that supports the conclusions. Experiments must have been conducted rigorously, with appropriate controls, replication, and sample sizes. The conclusions must be drawn appropriately based on the data presented.  Reviewer #1: Partly  Reviewer #2: Yes  Reviewer #3: Partly |  |  |  |
| 25 | 2. Has the statistical analysis been performed appropriately and rigorously?  Reviewer #1: Yes  Reviewer #2: Yes  Reviewer #3: No |  |  |  |
| 26 | 3. Have the authors made all data underlying the findings in their manuscript fully available? The [PLOS Data policy](http://www.plosone.org/static/policies.action#sharing) requires authors to make all data underlying the findings described in their manuscript fully available without restriction, with rare exception (please refer to the Data Availability Statement in the manuscript PDF file). The data should be provided as part of the manuscript or its supporting information, or deposited to a public repository. For example, in addition to summary statistics, the data points behind means, medians and variance measures should be available. If there are restrictions on publicly sharing data—e.g. participant privacy or use of data from a third party—those must be specified.  Reviewer #1: Yes  Reviewer #2: Yes  Reviewer #3: No |  |  |  |
| 27 | 4. Is the manuscript presented in an intelligible fashion and written in standard English? PLOS ONE does not copyedit accepted manuscripts, so the language in submitted articles must be clear, correct, and unambiguous. Any typographical or grammatical errors should be corrected at revision, so please note any specific errors here.  Reviewer #1: Yes  Reviewer #2: Yes  Reviewer #3: Yes |  |  |  |
|  | Reviewer #1: |  |  |  |
| 28 | In the Introduction section, the article describes the current status of the prevalence of micronutrient deficiencies worldwide and in India. This is a good point, however, the reason for focusing only on children and adolescents in urban areas is not given later in this section. It is recommended to add the appropriate content. | It is now written in the manuscript as “Vitamins and minerals, termed together as micronutrients, although required in small quantities, are vital for the normal growth, development and functioning of human body. These are essential throughout the life but the period of childhood and adolescence is more important, as it is marked by rapid growth and development. Review of literature suggests that deficiency of minerals (calcium, iron, selenium and zinc) and Vitamins (Vitamin A, 25 Hydroxy Vitamin D, Vitamin B12 and Folate) during childhood and adolescence have negative impact on general health, growth, neuropsychological behavior, cognitive and motor development, intelligence quotient, attention, learning, memory, language ability and educational achievement [1-9]. Iron, folate or vitamin B12 deficiency results in anaemia which has a negative impact on work capacity, intellectual performance, and child cognitive development [1]. Vitamin A plays a critical role in eye health and immune function [1]. Zinc deficiency can interfere with multiple organ systems including brain development and cognition, particularly when it occurs during a time of rapid growth and development [2].” | 3 | 58-70 |
| 29 | In the Data collection of the methodology section, the article does not list the specific demographic and socioeconomic data collected. Please consider adding related descriptions in this section. | Done as advised. It is now written as “To use the nested structure of children within school, multilevel logistic models as well as individual level logistic model were used to find the associations of important factors (sociodemographic profile like age, gender, family type, mother’s education, socioeconomic class and anthropometric indicators like severe thinness/thinness, overweight, obese/severe obese, stunting) on outcome variable (micronutrient deficiency).” | 9 | 201-205 |
| 30 | The article applied univariate and multivariate analysis to find the associations of important factors (sociodemographic profile and anthropometric indicators) on outcome variable (micronutrient deficiency) in addition to assessing the prevalence of micronutrient deficiencies.  However, these were not presented in the abstract and study objectives.  Moreover, the presentation of the logistic model is missing in the Methodology section as well as the specific results of the logistic analysis in the Results section.  In addition, the discussion section also lacks an in-depth discussion of the relevant content. It is recommended that these contents be supplemented. | We agree with esteemed reviewer. We have now written it as “The primary objective of study was to assess the prevalence of deficiencies of Vitamins (Vitamin A, 25 Hydroxy Vitamin D, Vitamin B12 and Folate) and minerals (Calcium, Zinc, Selenium and Iron), among urban school going children aged 6-11 and 12-16 years in ten cities of India. Our secondary objective was to find the association between micronutrient deficiencies with sociodemographic and anthropometric indicators”.  We have now written it as “To use the nested structure of children within school, multiple logistic regression model as well as individual level logistic model were used to find the associations of important factors (sociodemographic profile like age, gender, family type, mother’s education, socioeconomic class and anthropometric indicators like severe thinness/thinness, overweight, obese/severe obese, stunting) on outcome variable (micronutrient deficiency)”, as mentioned in point no 29. | 2  4 | 32-36  88-93 |
| 31 | The discussion section lacks a discussion of the limitations of the study. In addition, the discussion generally revolves around the findings of the prevalence of micronutrient deficiencies. I think this is a good point. However, the discussion is generally illogical and does not provide targeted guidance on how to improve micronutrient deficiencies in children and adolescents in urban India based on the findings of this article. Please consider reorganizing the discussion section to improve this section. | It is now written as “The present study is first of its kind, estimated the prevalence and magnitude of the problem, at country level. However, the current study was limited to the urban population. Due to logistic issues, we equally divided the total sample size across all ten districts, this may have possibly under-represented the districts with higher population densities. A selection bias was also possible as the current study included the participants residing within 5 km radius of the school.” | 16 | 346-350 |
| 32 | Abstract Please add the statistical methods used in the article in the Methodology section of the abstract. | It is now written as “Point estimates and 95% confidence intervals was used to assess the prevalence of deficiencies. Associations were observed using chi square, student t test and ANOVA test.” | 2 | 41-43 |
| 33 | The Introduction section lacks a specific description of existing relevant studies, which cannot well reflect the shortcomings of the current studies and highlight the research strengths of this study. Please consider adding related descriptions in this introduction. | It is now written as “National surveys in India, like National Family Health Survey 4 (2014-15) [12] reported high prevalence of amaemia among children aged 6 to 59 months, women aged 15 to 49 years (≥ 50%) and 22.7% among men aged 15 to 49 years. The National Nutrition Monitoring Bureau [13] reported 67 % to 78 % prevalence of anaemia among preschool children, adolescent girls, pregnant and lactating women in rural areas. These surveys including Coverage Evaluation Survey (2009) [14], Annual Health Survey (2012-13) [15], District Level Household Survey (2012-13) [16] had evaluated the nutritional status based on anthropometric measurements, dietary intake and presence of anemia [17, 18]. These surveys did not assess the micronutrient status, except National Nutrition Monitoring Bureau (2012), which reported prevalence of Vitamin A deficiency as 0.2%, on basis of Bitot’s spots. Many surveys to assess the burden of various micronutrient deficiencies were limited to state or district (19 - 29). Hence, there is scarcity of a nationally representative data on micronutrient status of children and adolescents” in the Introduction section.  Strengths and limitations of current study as mentioned in discussion section, | 4  16 | 74-85  346-350 |
| 34 | Line 71: Please add the corresponding references to this section. | Done as advised by esteemed reviewer. Reference numbers 19 -29. | 4 | 80 |
| 35 | Line 89-95: The existing descriptions in the “Study setting” section do not seem to give a good representation of the 10 cities. In the article, “Population aged 6 to 16 years was 7.1 million in these cities.”  What percentage of the population aged 6-16 in these ten cities accounts for the total population aged 6 to 16 in the country? Please give the corresponding values. | The study sites were located in central (Lucknow, Patna), northern (Srinagar, Chandigarh), southern (Bangalore, Bhubaneswar, Manipal and Thiruvananthapuram), eastern (Dibrugarh) and western (Jodhpur) parts of the country.  5.2% of India’s urban population aged 6 to 16 years reside in urban areas of these 10 cities.  This is now written as “Urban population aged 6 to 16 years was 4 million in these cities, which accounts to 5.2% of country’s population in this group”. | 5 | 110-111 |
| 36 | Line 97-98: Please give the formula for calculating the sample size and explain why the folic acid deficiency rate was assumed in the calculation of the sample size. | The formula used is the standard one “Sample size for prevalence with a given precision” as follows:  $n=\frac{z_{1-\alpha/2}^{2}}{d^{2}}$  Here: p = 0.307, d= 0.02, and α = 0.05.  We have calculated the sample sizes for other micronutrient deficiencies also and have picked the one (corresponding to folate deficiency) with maximum sample size. |  |  |
| 37 | Line 115-116: Please give the reference for the threshold “BMI <12.5%” as an exclusion criterion. In addition, please give the threshold values and references for the four categories of BMI for age in the corresponding section of the methodology. | Detailed study protocol is published elsewhere (Ref. no. 30) where the threshold values and references for the four categories of BMI for age is given. |  |  |
| 38 | Please be consistent with the term “folate” throughout the article. “Folate” or “folic acid”? | Done as advised by esteemed reviewer. |  |  |
| 39 | Please add the definition of “Stunted” in the Methodology. | Detailed study protocol is published elsewhere (Ref. no. 30) where the definition of stunted and other anthropometric parameters is given. |  |  |
| 40 | Line 251-252, Line 267: Please add the corresponding references to this section. | Done as advised by esteemed reviewer. |  |  |
| 41 | In the discussion, the authors repeatedly compare their main results with the National Comprehensive Nutrition Survey (CNNS). In my opinion, if these survey data from the CNNS are so important, it is recommended that a brief description of the survey-related elements of the CNNS, such as the survey population and survey time, be given in the article, along with the corresponding references. | Done as advised by esteemed reviewer. It is now written as “India is also facing the challenge of micronutrient malnutrition as reported by data from Comprehensive national nutrition survey (CNNS) [18] which was published in 2019, at the time when the current study was operationalized. CNNS lacks the data about calcium and selenium status of population.” | 12 | 249-252 |
| 42 | Conclusion Based on the main results of the article, it seems inappropriate to conclude that “with strengthening the existing health and agricultural system and proper implementation of food fortification guidelines, we may expect improvement in the micronutrient status of population in coming years”. Please verify and be cautious in drawing conclusions based on the main findings of the article. | It is now written as “The present study comprehensively provides information on the status of micronutrient deficiencies in Indian school-going children and adolescents. Almost half of the population was deficient in calcium and iron and one third were having deficiency of 25 hydroxy Vitamin D and Vitamin B12. One or more micronutrient deficiencies were found in almost one half of study population.” | 16 | 351-356 |
|  | Reviewer #2: |  |  |  |
| 43 | Present some data on the national surveys mentioned between lines 66-68. | Done as advised. Reference numbers 12 – 16. | 4 | 74-79 |
| 44 | Between lines 73-78, it seems the informations is repeated. | Done as advised by esteemed reviewer. It is now written as “The aim of this current study is to fill this knowledge gap by collecting data through a well-planned, large-scale study using standardized methodologies to assess the prevalence of various micronutrient deficiencies, among urban school going children. The primary objective of study was to assess the prevalence of deficiencies of Vitamins (Vitamin A, 25 Hydroxy Vitamin D, Vitamin B12 and Folate) and minerals (Calcium, Zinc, Selenium and Iron), among urban school going children aged 6-11 and 12-16 years in ten cities of India. Our secondary objective was to find the association between micronutrient deficiencies with sociodemographic and anthropometric indicators.” | 4 | 86-93 |
| 45 | Why the choice of using folate in the sample size calculation. | We have calculated the sample sizes for other micronutrient deficiencies also and have picked the one (corresponding to folate deficiency) which gave maximum sample size. This has now been written in the manuscript as “The sample size was calculated using folate deficiency as this gave the maximum sample. Assuming the prevalence of folate deficiency in India as 30.7% [32], precision (d) of 2% and level of significance (α) 0.05, the calculated sample size [33] was 2044 participants. After taking, attrition rate of 10% sample size inflate to 2400 participants.” | 5 | 113-116 |
| 46 | Is table 3 really necessary? I find it is not adding much to the manuscript | Removed as advised by esteemed reviewer. |  |  |
| 47 | Why the difference in type of family and the implications in results was not discussed? | Difference in type is removed, as advised by other reviewer. |  |  |
| 48 | Between lines 230-239, can you present some data comparisons with other countries? | Done as advised by reviewer. It is now written as “Globally, an estimated 29% of preschool children have vitamin A deficiency, 18% have anemia and 17% of the population are at risk of inadequate zinc intake.” | 12 | 248-249 |
| 49 | Why results of crossing BMI with micronutrient deficiencies was not discussed? | This is now added. It is now written as “In our study, we found statistically significant difference across the BMI categories of participants having calcium deficiency. The proportion of participants with calcium deficiency was higher in severely thin/thin category. A study from Indonesia, reported that level of calcium and vitamin D were not associated with the incidence of stunting [72]. Another study on Ecuadorian children reported that low serum vitamin D levels in underweight and stunted [73].  We also observed statistically significant difference across the various BMI categories in the participants having Zinc and selenium deficiency. The higher proportion of participants with zinc deficiency were found to be overweight, obese / severely obese. Similar to our findings, Gu et al in their meta-analysis found that serum zinc level was significantly lower in obese children [74]. Many other researchers also reported decrease in zinc levels in overweight, obese and severely obese [75, 76]. Decreased selenium levels are also reported in overweight, obese / severely obese children [77], complementing to our findings (table 4).” | 16 | 334-345 |
| 50 | Most participants were from public schools, did it not implicated in the results? | Our sampling frame consists of schools within the urban boundary of the city. We had considered government aided schools as Government schools only. There were more government schools than private schools. Selection of schools was done in similar ratio. Hence our sample has more participants from public/government schools.  This has now been added in the methods section. It is now written as “Each study district provided a list of schools imparting co-education to children between 6 to 16 years of age and located within the urban limits of city. From this list, six schools were selected using simple random sampling by GGA, having at least one to a maximum of three private schools. The government aided schools were considered as government schools only. There were more government schools than private schools. Selection of schools was done in similar ratio.” | 5-6 | 120-125 |
| 51 | Please add the study limitations | Done as advised. It is now written as “However, the current study was limited to the urban population. Due to logistic issues, we equally divided the total sample size across all ten sites, this may have possibly under-represented the sites with higher population densities”. | 16 | 346-350 |
|  | Reviewer #3: | | | |
| 52 | Title and all through the text (e.g. abstract, methods) - I think you mean "multicentre" and not "multicentric". | Done as advised by esteemed reviewer. |  |  |
| 53 | Abstract line 44-45: I believe the percentages in brackets are the proportions of participants who were deficient of the named vitamins. The statement says that there were deficient in more than one quarter of subjects, but for folate the prevalence of deficiency 22.2% is less than a quarter. | Done as advised by esteemed reviewer. It is now written as “25 Hydroxy Vitamin D deficiency was seen in 39.7% and vitamin B12 in 33.4% of subjects. Folate, Selenium and Zinc were deficient in 22.2%, 10.4% and 6.8% of subjects respectively.” | 2 | 47-49 |
| 54 | line 82: you should name the approving institutional ethics committee and include the approval reference number and date where available. | This information is given under the section “Ethical approval and consent to participate”, after conclusion. | 16-17 | 357-368 |
| 55 | study design - it would be useful to provide more details about how the sites/schools were selected; it is not sufficient to simply say that they were randomly selected. For example, how was the random selection list generated? How did you ensure that the schools selected were representative of the population? etc. | The detailed study protocol is now published elsewhere (Ref. no. 30). |  |  |
| 56 | This point needs to be further addressed in light of the decision to equally divide the total sample size across all ten sites - this would mean that sites whose catchment areas had higher population densities were under-represented in the sample, since they contributed the same number of individuals to the sample as less population dense areas. | This is prerequisite of multicentric study to take approximately equal sample size. Otherwise very difficult to plan multicentric study. Cities were not selected on the basis of density. They were selected so as to cover the population as a whole. The representativeness of the population was taken care by the process of subject selection. |  |  |
| 57 | in selection of participants you indicate that this was a two-stage sample; you need to further describe how the sampling in each stage was conducted: for example, you indicate that the first stage sampling included 1-3 private schools - how did you ensure this, given that you have no control of this in a random sampling; how was the random selection done for the second stage? The method of random selection should be described. | The detailed study protocol is now published elsewhere (Ref. no. 30). |  |  |
| 58 | Additionally, I think you should replace "site" with "district", as the sites were actually the schools. | Done as advised. |  |  |
| 59 | statistical analysis - you state in line 178 that chi-squared tests were used for comparison of proportions, in line 180-181 that t-tests and ANOVA were used to compare means, and in lines 182-185 that logistic regression models were used to explore associations; however, there is nothing in the objectives of the study calling for any comparisons between groups or exploration of associations. If the study aimed to estimate prevalences of micronutrient deficiency, then these analyses were probably pointless. What would be useful to report would be methods for calculating the prevalences that took into account the clustered nature of the data. | Our primary study objective was “The objective of study was to assess the prevalence of deficiencies of Vitamins (Vitamin A, 25 Hydroxy Vitamin D, Vitamin B12 and Folate) and minerals (Calcium, Zinc, Selenium and Iron), among urban school going children aged 6-11 and 12-16 years in ten cities of India”.  It clearly states that we will be comparing the results between the two groups i.e. 6-11 years and 12-16 years. |  |  |
| 60 | Results - related to the comment above, the column of p-values in Tables 4 and 5 (and any commentary about these) are unnecessary and should be removed, as they don't address any of the objectives of the study (i.e. the study was not set up to investigate whether group 1 and group 2 differed in terms of characteristics and outcomes - if it was then this would be the wrong design for such a study). In any case, this table is supposed to be purely descriptive of the sample included in the analysis. | p-values removed in from table 2 and 3 (erstwhile table 4 and 5 respectively). |  |  |
|  | Furthermore the commentary in lines 209-211 do not really address any clear aims of the study. The analyses comparing micronutrient deficiency between males and females, across different age groups, and across BMI are exploratory and should be presented as such. | This corresponds to the secondary objective, which is now written as “Our secondary objective was to find the association between micronutrient deficiencies with sociodemographic and anthropometric indicators”. | 4 | 91-93 |
| 61 | PLOS authors have the option to publish the peer review history of their article ([what does this mean?](https://journals.plos.org/plosone/s/editorial-and-peer-review-process#loc-peer-review-history)). If published, this will include your full peer review and any attached files. If you choose “no”, your identity will remain anonymous but your review may still be made public. **Do you want your identity to be public for this peer review?** For information about this choice, including consent withdrawal, please see our [Privacy Policy](https://www.plos.org/privacy-policy).  Reviewer #1: No  Reviewer #2: **Yes:** Giselle Rhaisa do Amaral e Melo  Reviewer #3: No |  |  |  |
